# Whole-Body Electromyostimulation Coupled with Aerobic Exercise Boosts Serum Irisin Levels in Healthy Individuals: A Pilot Study

**DOI:** 10.3390/jfmk10030308

**Published:** 2025-08-08

**Authors:** Gianluca Vadalà, Giuseppina Di Giacomo, Fabrizio Russo, Veronica Tilotta, Raffaella Rosy Vescio, Claudia Colaiacomo, Giorgia Petrucci, Luca Ambrosio, Vincenzo Denaro, Rocco Papalia

**Affiliations:** 1 Operative Research Unit of Orthopaedic and Trauma Surgery, Fondazione Policlinico Universitario Campus Bio-Medico, 00128 Rome, Italy; g.vadala@policlinicocampus.it (G.V.); g.digiacomo@unicampus.it (G.D.G.); v.tilotta@policlinicocampus.it (V.T.); raffaella.vescio@unicampus.it (R.R.V.); claudia.colaiacomo@unicampus.it (C.C.); g.petrucci@policlinicocampus.it (G.P.); l.ambrosio@policlinicocampus.it (L.A.); denaro@policlinicocampus.it (V.D.); r.papalia@policlinicocampus.it (R.P.); 2 Research Unit of Orthopaedic and Trauma Surgery, Departmental Faculty of Medicine and Surgery, Università Campus Bio-Medico di Roma, 00128 Rome, Italy

**Keywords:** electromyostimulation, exercise, exerkine, irisin, joints, myokines, osteoarthritis

## Abstract

**Background:** Irisin, a myokine secreted during physical activity, has garnered attention for its potential roles in cartilage homeostasis and musculoskeletal health. Whole-body electromyostimulation (WB-EMS) is an emerging exercise modality that enhances muscle recruitment and may stimulate greater irisin release. This study aimed to compare the acute serum irisin response following aerobic exercise with and without WB-EMS in healthy individuals. **Methods**: A total of 24 healthy adults were enrolled and randomized to undergo either aerobic physical activity (PA) or WB-EMS (n = 12 each). Both groups performed identical exercise routines, with the WB-EMS group additionally receiving muscle stimulation via a standardized protocol. Serum irisin levels were measured at baseline (T0), 10 minutes post-exercise (T1), and 1 hour post-exercise (T2) using ELISA. Statistical analysis was performed using two-way ANOVA with post hoc testing. **Results**: At T1, serum irisin levels did not significantly differ from baseline in either group. At T2, the WB-EMS group demonstrated a statistically significant increase in irisin levels compared to both T0 and T1 (*p* < 0.01), as well as to the PA group (*p* < 0.01). In contrast, the PA group showed only a slight, non-significant rise at T2. These findings suggest that WB-EMS induces a more robust irisin response than traditional aerobic exercise. **Conclusions**: WB-EMS appears to enhance irisin release in healthy individuals following acute exercise. These results support further research into WB-EMS as a novel strategy to modulate myokine production with potential therapeutic relevance in musculoskeletal conditions such as osteoarthritis.

## 1. Introduction

Physical activity (PA) exerts profound effects on the musculoskeletal system, promoting muscle hypertrophy, enhancing bone mineral density, and preserving joint integrity through both mechanical loading and molecular signaling. Regular exercise stimulates osteogenic and chondrogenic pathways, increases synovial fluid circulation, and attenuates pro-inflammatory mediators that contribute to cartilage degradation and subchondral bone remodeling [1]. In addition to these biomechanical benefits, PA induces a systemic anti-inflammatory response by modulating cytokine profiles, decreasing circulating levels of tumor necrosis factor-α (TNF-α) and interleukin-6 (IL-6), and upregulating anti-inflammatory mediators such as interleukin-10 (IL-10). Collectively, these effects help maintain joint homeostasis, improve functional capacity, and reduce pain in both healthy individuals and those with degenerative musculoskeletal disorders, such as osteoarthritis (OA) [2].

Beyond mechanical effects, the skeletal muscle releases a variety of bioactive factors collectively termed “exerkines” that exert autocrine, paracrine, and endocrine actions on different tissues, including the bone, cartilage, and tendon [3]. Among these, irisin has emerged as a particularly promising myokine. Identified in 2012 by Boström et al., irisin is cleaved from fibronectin type III domain-containing protein 5 (FNDC5) in response to PGC-1α activation during muscle contraction. It is involved in various physiological processes, including browning of white adipose tissue, increased energy expenditure, and anti-inflammatory effects [4,5,6]. Recent evidence suggests that irisin may also contribute to musculoskeletal health and cartilage regeneration [7]. Our research group was the first to demonstrate that irisin exerts anabolic effects on human articular chondrocytes, enhancing extracellular matrix production and mitigating osteoarthritic degeneration in vitro [8]. These findings support further investigation into irisin as both a biomarker and a therapeutic agent in OA.

Given the potential of irisin to mediate beneficial cross-talk between the skeletal muscle and joint tissues, there is considerable interest in identifying exercise modalities that could maximize its release. In this context, innovative exercise modalities such as whole-body electromyostimulation (WB-EMS) have gained attention, especially for individuals with limited capacity for conventional exercise. WB-EMS delivers low-frequency electrical impulses, leading to the simultaneous stimulation of large muscle groups [9]. Mechanistically, electrical stimulation enhances skeletal muscle cell metabolism by upregulating AMPK, JNK, and AKT pathways, and increasing the expression of GLUT4 and the irisin precursor PGC-1α [10]. Furthermore, EMS has also been shown to promote satellite cell viability [11], stimulate local osteogenesis [12], and increase muscle mass and function [13]. Emerging evidence supports its efficacy in improving muscle quality and metabolic health even in older or sedentary populations. Although generally safe, cases of rhabdomyolysis have been reported following WB-EMS training, especially in frail individuals or after unsupervised, excessive electrical stimulation [14].

Recently, a randomized controlled trial of 72 overweight patients with knee OA showed that WB-EMS significantly reduced joint pain and improved daily function, mobility, muscle strength, and quality of life compared to standard care [15]. These findings underscore the potential of WB-EMS as an adjunct or alternative intervention for patients unable or unwilling to engage in traditional exercise programs [16].

We hypothesize that PA, particularly when augmented by WB-EMS, may further stimulate muscle contraction, thus elevating systemic and local intraarticular irisin levels, thereby possibly exerting protective effects on joint tissues. The objective of this pilot study was to investigate the differential serum irisin changes following aerobic PA with vs. without WB-EMS in a small cohort of healthy individuals.

## 2. Materials and Methods

This pilot study was conducted at Fondazione Policlinico Universitario Campus Bio-Medico (Rome, Italy) and was approved by the local Institutional Review Board under the ID n. 45.24EM.CET2 on 16 April 2025. All participants were identified and provided written informed consent before enrolment in the presence of research staff members directly involved in the study.

### 2.1. Study Population

**Figure 1:**
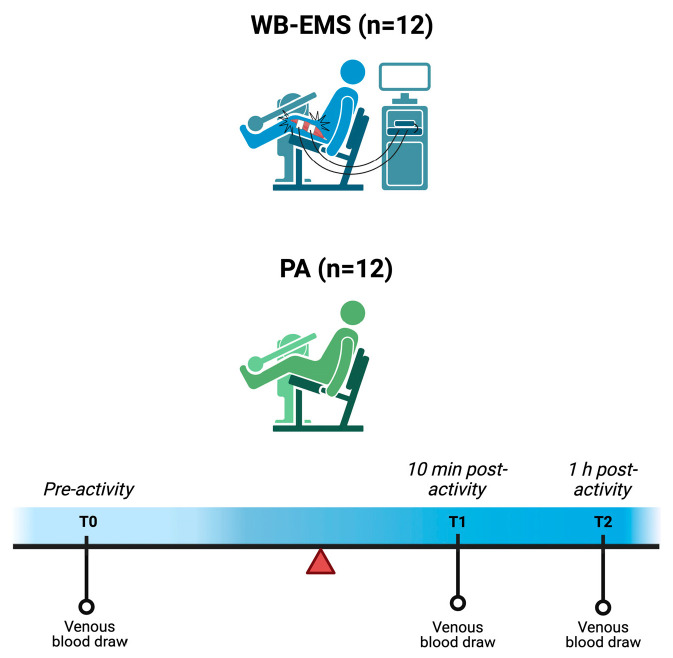
Schematic representation of the study design. Abbreviations: PA = physical activity, WB-EMS = whole-body electromyostimulation. Created with BioRender.com.

Eligible participants were healthy adults between 20 and 70 years of age, with no known medical conditions. Specific exclusion criteria included the presence of a pacemaker, pregnancy, or a history of severe cardiovascular, respiratory, renal, or neurological disorders, or any somatic or clinical contraindications to wearing WB-EMS equipment, abdominal or inguinal hernia, malignancy, diabetes mellitus, haemophilia, febrile illnesses, or acute bacterial or viral infections. Participants were also excluded if they reported either a completely sedentary lifestyle or engagement in professional athletic training, in order to avoid extremes of physical activity that could bias irisin blood measurements. Twenty-four healthy, WB-EMS-naïve, adult volunteers were enrolled in the study without financial compensation. Each participant was registered in a secure database containing demographic information (surname, first name, sex, date and place of birth, and occupation), lifestyle characteristics (contraceptive use, smoking, and alcohol consumption), and contact details (address and phone numbers). Once eligibility was confirmed, all participants underwent physical examination and venous blood sampling. Laboratory analyses included complete blood count, serum sodium, chloride, and potassium levels, liver enzymes (aspartate aminotransferase [AST] and alanine aminotransferase [ALT]), blood urea nitrogen, creatinine, prothrombin time (PT), activated partial thromboplastin time (APTT), blood glucose, myoglobin, creatine kinase (CK), C-reactive protein (CRP), and irisin levels. For women of reproductive age, a pregnancy test was also performed. Following recruitment, the study participants were equally divided and randomly assigned to either of two intervention groups through 1:1, simple, computer-generated randomization. The study design is depicted in Figure 1.

### 2.2. Intervention

#### 2.2.1. WB-EMS Protocol

Participants in the WB-EMS group (n = 12) followed a supervised exercise protocol while wearing a WB-EMS suit (Justfit System, Justfit Technology Ltd., Budapest, Hungary) in the presence of a certified personal trainer not involved in the study. The EMS protocol was performed using a device delivering bipolar electrical stimulation at a fixed frequency of 85 Hz, with 4 s of stimulation followed by 4 s of rest, 350 μs pulse width, and a 0.4 s ramp time. These parameters have been shown to be safe, with a very low risk of inducing rhabdomyolysis [16]. Surface electrodes were positioned on the upper and lower limbs as well as the torso. Each session began with a 10 minute-warm-up on a cycle ergometer, after which participants wore the WB-EMS suit. The exercise component of the session involved a series of bodyweight movements targeting the lower limbs and core muscles. Each EMS session lasted 20 minutes and was structured around a series of functional exercises, including: half squat, squat with chest fly, shoulder external rotation, crunch, front squat, pull-up, oblique crunch, lunge, lateral raise, and bent-over row with triceps extension. Exercise type, intensity, and difficulty were individually tailored to match each participant’s physical fitness and endurance levels.

#### 2.2.2. Aerobic PA Protocol

Participants in the PA group (n = 12) followed the same exercise protocol as the WB-EMS group, but without the application of the WB-EMS equipment. This approach ensured that both groups engaged in equivalent PA regimens, with the only variable being the use of WB-EMS technology.

### 2.3. Blood Sample Collection

All participants observed a fasting period starting three hours before the initial blood collection (T0) and remained fasting throughout the physical exercise session, regardless of group assignment (PA or WB-EMS). For analysis, 5 mL of venous blood were collected from each participant at three time points: T0 (pre-activity), T1 (10 minutes post-activity), and T2 (1 hour post-activity). These sampling time points were applied uniformly across both groups. All exercise sessions were performed in the morning. Samples were centrifuged at 4000 rpm for 5 minutes and stored at −80 °C until analysis.

### 2.4. Assessment of Serum Irisin Levels

Irisin serum levels were measured with a commercial ELISA kit (Irisin recombinant Elisa Protocol, EK-067-29, Phoenix Pharmaceuticals, Schiltigheim, France). The limit of sensitivity was fixed at 1.29 ng/mL. Measurements were performed according to the manufacturer’s instructions, with samples diluted to 1/10. All assays were performed in duplicate, and a positive control validated the experimental conditions. Absorbance was read at 450 nm using a Tecan Infinite M200 PRO Plate Reader.

### 2.5. Sample Size and Statistical Analysis

With 12 participants per group (24 total), a two-sided α of 0.05, and an anticipated fold-change standard deviation (SD) of 0.30, the study achieved 80% power to detect a between-group difference of at least 0.343 ng/mL. Data were expressed as absolute frequencies (n) and relative percentages or means ± SD. The normality of data distribution was assessed using the Wilk–Shapiro test. Based on the type of data, formal analysis was performed using Fisher’s exact test, unpaired *t*-test, or two-way ANOVA applying a mixed-effects model and Fisher’s least significant difference test for multiple comparisons. The value of *p* < 0.05 was considered statistically significant. Formal analysis was performed using the GraphPad Prism 10.2.0 software (Dotmatics, Boston, MA, USA).

## 3. Results

The WB-EMS group (n = 12; 9 males, 3 females) had a mean age of 26.6 ± 3.1 years (range: 23–31 years) and a mean BMI of 22.6 ± 2.8 kg/m^2^, while the PA group (n = 12; 7 males, 5 females) had a mean age of 26.9 ± 2.9 years (range: 24–29 years) and a mean BMI of 22.2 ± 3.0 kg/m^2^. Upon recruitment, laboratory values fell within normal ranges in all study participants (Table 1). Baseline demographic characteristics and laboratory values were comparable, with no significant intergroup differences.

At baseline (T0), irisin level was 17.41 ± 5.13 ng/mL in the WB-EMS and 15.69 ± 6.25 in the PA group (*p* > 0.05). At T1 (10 minutes post-exercise), serum irisin levels in both groups remained similar to T0 (WB-EMS: 0.98 ± 0.30; PA: 1.09 ± 0.27), with no significant differences observed.

**Figure 2:**
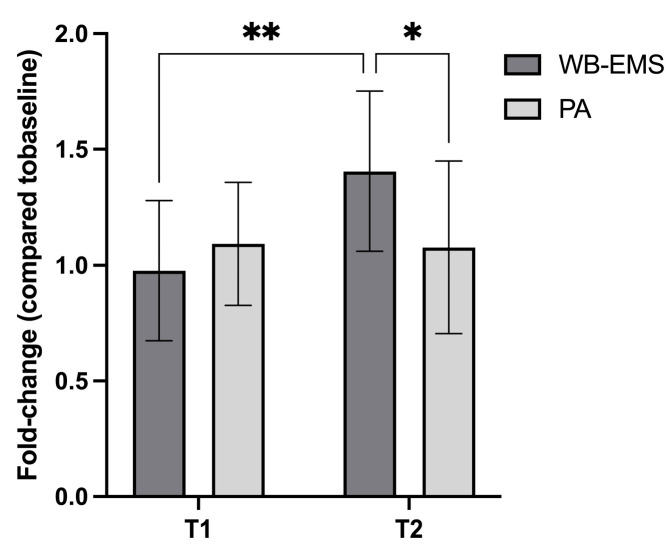
Serum irisin levels (normalized to baseline, T0 = 1) measured at two time points (T1 and T2) following two interventions: whole-body electromyostimulation (WB-EMS) and physical activity (PA). At T2 (1 h post-intervention), the WB-EMS group exhibited a significantly greater increase in irisin concentration compared to both its baseline and the PA group. Within the WB-EMS group, data from one subject at T1 and two subjects at T2 were excluded due to fast-breaking, resulting in an n = 11 and n = 10 at T1 and T2, respectively. Statistical significance: * *p* < 0.05, ** *p* < 0.01.

However, a differential response emerged at T2 (1 hour post-exercise). The PA group exhibited only a slight, non-significant increase in irisin levels (1.08 ± 0.37), whereas the WB-EMS group showed a significantly greater elevation (1.41 ± 0.34) compared to both baseline and T1. Furthermore, at T2, the increase in irisin levels in the WB-EMS group was significantly higher than in the PA group, indicating that WB-EMS served as a more potent stimulus for irisin release than conventional PA (Figure 2). No adverse events occurred during any of the experimental exercise activities.

## 4. Discussion

Irisin is a myokine released predominantly in response to muscle contraction, and it plays a key role in energy metabolism, inflammation regulation, and potentially cartilage homeostasis. Emerging evidence suggests that irisin may exert protective and regenerative effects on joint tissues, making it a promising target in musculoskeletal health research [8,17,18,19,20]. In this study, we preliminarily showed that WB-EMS induces a significantly greater increase in circulating irisin levels compared to traditional aerobic PA, when measured one hour post-intervention in healthy individuals. These results suggest that WB-EMS acts as a more potent physiological stimulus for irisin release, potentially offering enhanced biological effects relevant to musculoskeletal function and joint integrity. Previous studies have also shown that the addition of EMS to PA can further stimulate the release of anabolic factors such as cortisol and growth hormone following acute exercise [21]. We may speculate that these hormones might exert an additive effect together with irisin, thus further promoting post-exercise endurance adaptation and muscle anabolism.

However, it is noteworthy that irisin regulation under normal physiological conditions remains unclear. In healthy individuals, circulating irisin levels are subject to considerable interindividual variability and are influenced by a range of factors, including metabolic status, obesity, physical exercise or sedentary behavior [18]. Despite this complexity, our findings support the potential effectiveness of WB-EMS as a non-pharmacological intervention capable of modulating irisin release, at least in healthy subjects. Indeed, it is possible that similar results may not be observed in other populations, such as individuals with sarcopenia, reduced exercise tolerance, or comorbidities, due to factors like lower muscle mass, inadequate muscle activation, or altered metabolic responses. Further longitudinal and mechanistic studies are warranted to confirm and broaden the external validity of the present findings, clarify the biological pathways involved, and evaluate the clinical relevance of irisin secretion following WB-EMS, particularly in patients with osteoarthritis or reduced exercise tolerance.

## 5. Conclusions

In this pilot study, WB-EMS coupled with aerobic exercise elicited a significantly greater post-exercise increase in circulating irisin compared to conventional aerobic exercise. These findings highlight the potential of WB-EMS as a potent stimulus for myokine release and a promising non-pharmacological approach to support joint health and musculoskeletal function. Further research is warranted to explore its applications in clinical populations.

## Figures and Tables

**Table 1:** Laboratory values of the study participants.

Parameter (Reference Range)	WB-EMS	PA
WBC (4–10 × 10^3^/µL)	7.08 ± 1.24	7.20 ± 1.34
Hb (M: 13.5–17.5 g/dL; F: 12–16 g/dL)	15.04 ± 1.18	15.16 ± 1.32
Serum sodium (136–145 mmol/L)	138.40 ± 2.14	138.07 ± 2.01
Serum chloride (98–107 mmol/L)	102.14 ± 2.97	101.60 ± 2.76
Serum potassium (3.50–5.10 mmol/L)	4.09 ± 0.34	4.12 ± 0.33
AST (5.34–30.00 U/L)	25.93 ± 6.78	24.70 ± 5.14
ALT (0.00–55.00 U/L)	23.79 ± 6.47	23.10 ± 6.05
Urea (21.4–42.8 mg/dL)	30.04 ± 7.25	29.11 ± 6.81
PT (9–14 s)	13.39 ± 0.55	15.19 ± 2.71
APTT (27.50–41.50 s)	33.12 ± 3.36	34.19 ± 3.70
Blood glucose (70–99 mg/dL)	85.43 ± 6.96	84.30 ± 5.58
Myoglobin (M: 0–154 ng/mL; F: 0–106 ng/mL)	36.36 ± 23.73	41.30 ± 33.55
CK (29–168 IU/L)	101.57 ± 23.73	106.00 ± 25.69
CRP (<0.50 mg/dL)	0.27 ± 0.10	0.24 ± 0.13
Serum creatinine (M: 0.73–1.18 mg/dL; F: 0.55–1.02 mg/dL)	0.89 ± 0.15	0.97 ± 0.16

Abbreviations: ALT = alanine transaminase; APTT = activated partial thromboplastin time; AST = aspartate transaminase; CK = creatine kinase; CRP = C-reactive protein; Hb = hemoglobin; PT = prothrombin time; WBC = white blood cells. Values are expressed as mean ± standard deviation.

## Data Availability

The data presented in this study are available on reasonable request from the corresponding author.
